# Triglyceride/high-density cholesterol ratio as a predictor of cardiometabolic risk in young population

**DOI:** 10.25122/jml-2024-0117

**Published:** 2024-07

**Authors:** Claudia Veronica Mederos-Torres, Yolanda Díaz-Burke, María Luisa Muñoz-Almaguer, Alejandra Guadalupe García-Zapién, Rosario Lizette Uvalle-Navarro, Claudia Elena González-Sandoval

**Affiliations:** 1 Department of Pharmacobiology, University Center for Exact Sciences and Engineering, University of Guadalajara, Guadalajara, Mexico; 2 Academic Body UDG-CA-156 Biomedical Science and Toxicology, University of Guadalajara, Guadalajara, Mexico

**Keywords:** TG/HDL ratio, obesity, insulin resistance, young people

## Abstract

Cardiovascular disease remains a leading cause of morbidity and mortality worldwide. Understanding and detecting risk factors are crucial for early diagnosis and prevention strategies. Obesity, dyslipidemia, hypertension, and insulin resistance, among others, have been described as modifiable risk factors. Among these, the triglycerides-to-HDL cholesterol (TG/HDL) ratio has been described as a marker of insulin resistance and a predictor of cardiovascular disease. Our objective was to investigate the association between the TG/HDL ratio and various cardiometabolic risk factors. A total of 239 young adults aged 18-24 years were recruited. We assessed anthropometric measurements, lipid profiles, glucose levels, insulin, the HOMA index, and the TG/HDL ratio. Participants were stratified based on their BMI and TG/HDL ratio. Our findings revealed that individuals with an elevated TG/HDL ratio had higher blood pressure, BMI, waist circumference, cholesterol, and triglyceride levels compared to those with a normal ratio. Specifically, the TG/HDL ratio was associated with an odds ratio (OR) of 9.3 for overweight, 27.5 for obesity, and 4.41 for abdominal obesity. Additionally, the HOMA index, which measures insulin resistance, was higher in those with an elevated TG/HDL ratio, with a prevalence of 45.6%. In conclusion, the TG/HDL ratio is a predictive marker of insulin resistance in young individuals and is associated with modifiable risk factors for cardiometabolic disease.

## INTRODUCTION

Cardiovascular disease (CVD) remains a leading cause of morbidity and mortality worldwide. Early detection and prevention strategies are critical for managing this major health issue, necessitating a thorough understanding of risk factors and reliable biomarkers [1]. The most frequent modifiable risk factors for CVD are obesity, hypertension, diabetes mellitus, dyslipidemia, smoking, and age — each contributing to the development and progression of atherosclerosis [2].

Obesity is currently recognized as a pandemic by the World Health Organization (WHO) and various international health associations [3]. Over the years, it has been defined as the excessive or abnormal accumulation of adipose tissue, with the body mass index (BMI) commonly used to classify individuals [4]. According to the WHO, a BMI between 25 and 29.9 Kg/m^2^ is considered overweight, while a value equal to or greater than 30 Kg/m^2^ is classified as obesity [3]. The factors associated with the development of this pathology are diverse, including genetic, environmental, and behavioral elements [5].

Epidemiological data reveal a dramatic increase in obesity worldwide, with rates tripling since 1975. In 2016, it was estimated that more than 1.9 billion adults aged 18 years or older were overweight, with 650 million classified as obese, accounting for 13% of the global population. [2]. According to the Global Obesity Observatory, in 2022, 74% of adults over 20 years of age in Mexico were overweight or obese, with 38% classified as overweight and 36 % as obese [6]. Obesity is associated with numerous chronic health conditions, such as hypertension, dyslipidemia, type 2 diabetes, insulin resistance, cardiovascular diseases, various types of cancer and even kidney disease [7]. Recent studies have highlighted its role in exacerbating infectious diseases, including influenza, rhinovirus, and parainfluenza. During the COVID-19 pandemic, obesity was found to increase the likelihood of hospitalization and adverse outcomes [8,9].

Among the associated diseases, diabetes and cardiovascular diseases have seen significant increases. In Mexico, cardiovascular disease has been the leading cause of death over the past five years (20.1%), followed by type 2 diabetes mellitus (T2DM) (15.2%) and malignant tumors (12.1%) [10].

Researchers have been investigating simple, effective markers to identify modifiable risk factors early. The triglyceride-to-high-density lipoprotein (TG/HDL) ratio has emerged as a useful predictor of visceral adipose tissue accumulation, insulin resistance (IR), and metabolic abnormalities associated with T2DM and cardiovascular disease [11,12]. The TG/HDL ratio has been associated with insulin resistance, hyperinsulinemia, obesity, metabolic syndrome, and the clear association between dyslipidemia and cardiovascular disease [13,14]. In particular, individuals with abdominal adiposity are at a higher risk for insulin resistance, elevated triglyceride levels, and low HDL cholesterol levels. The TG/HDL ratio effectively identifies these metabolic abnormalities [11,14]. In apparently healthy young people, this ratio has shown to be a good predictor of IR even in the absence of obesity, and high levels have been observed in obesity and dyslipidemias, so its use and application can be extended to promptly identify these disorders at an early age [15,16]. This study aimed to explore the relationship between the TG/HDL ratio and obesity, abdominal obesity, insulin resistance, and hypertension. By establishing guidelines for early detection of cardiometabolic risk, we seek to enhance preventive measures and improve health outcomes in the younger population.

## MATERIAL AND METHODS

A total of 239 students from the University of Guadalajara in Mexico, between 18 and 24 years old, were recruited. They were invited through an open call and were selected based on age (18-24 years) and a BMI greater than 19 kg/m^2^. The research was carried out following the guidelines of the Regulations of the General Health Law on Research in Mexico and the principles of the Helsinki Declaration 2008.

### Participant assessment

The selected participants completed a medical history questionnaire and underwent measurements of height, weight, and blood pressure, as well as blood sampling for biochemical determinations. Weight and height were measured using a TANITA30A scale, which automatically calculates the BMI. The BMI was then used to classify the population into normal weight, overweight and obese categories, with values of 19–24.9 kg/m^2^, 25–29.9 kg/m^2^, and ≥30 kg/m^2^ BMI, respectively [3]. Blood pressure measurements were conducted following the recommendations of NOM-030-SSA2-2009 using an aneroid sphygmomanometer. A cutoff point of 135/90 was considered for the diagnosis of hypertension (HT) [17].

### Biochemical analysis

Blood samples were collected and divided into two aliquots. One aliquot was used for determining glucose, cholesterol, triglycerides, and HDL levels using a dry chemistry technique with a FujiFilm Drichem NX500i. The TG/HDL ratio was calculated using the triglyceride and HDL results, with elevated ratios defined as ≥2.5 for women and ≥3.5 for men [12]. The second aliquot was used to measure insulin levels via an ELISA sandwich-type technique with a Bio-Rad Pre-Diabetes Human Assay Kit. Insulin resistance was assessed using the HOMA-IR index formula, with a cutoff point of >2.5 used to differentiate between individuals with and without insulin resistance [18].

### Statistical analysis

Descriptive statistics were calculated for all data, including frequencies, means, and standard deviations. The students’ t-test was used to compare the results between groups. The chi-square test was employed to assess associations between variables and to calculate odds ratios. All statistical analyses were conducted using Statgraphics Centurion 19.

## RESULTS

A total of 239 students from the University of Guadalajara participated in the study. Their general demographic, anthropometric, and biochemical characteristics are shown in Table 1. Gender differences in biochemical parameters such as glucose, total cholesterol, and triglycerides are highlighted, revealing higher levels in men.

**Table 1:** Description of anthropometric and biochemical variables by gender

Variable	Women *n* = 135	Men *n* = 104	*P*
Age (years)	20.05 ± 1.48	20.80 ± 1.70	
SBP (mmHg)	107.54 ± 9.63	118.48 ± 10.94	<0.000
DBP (mmHg)	71.34 ± 9.78	75.42 ± 10.38	0.000
WC (cm)	81.18 ± 7.14	93.11 ± 14.42	<0.000
BMI (Kg/m^2^)	24.5 ± 4.48	26.86 ± 6.01	0.001
Glucose (mg/dL)	81.22 ± 7.14	82.86 ± 7.17	0.073
Cholesterol (mg/dL)	169.09 ± 32.77	166.64 ± 64.76	0.577
Triglycerides (mg/dL)	89.80 ± 66.94	166.64 ± 34.76	0.000
HDL (mg/dL)	50.48 ± 12.53	41.82 ± 10.72	<0.000
TG/HDL	1.92 ± 1.60	3.19 ± 3.95	0.000
HOMA-IR	2.83 ± 2.00	3.26 ± 2.32	0.129

Mean ± standard deviation, *P* value obtained using Student’s t-test. SBP, Systolic Blood Pressure; DBP, Diastolic Blood Pressure; WC, Waist Circumference; BMI, Body Mass Index; HDL High-Density Lipoprotein; HOMA, Homeostatic Model Assessment.

**Figure 1:**
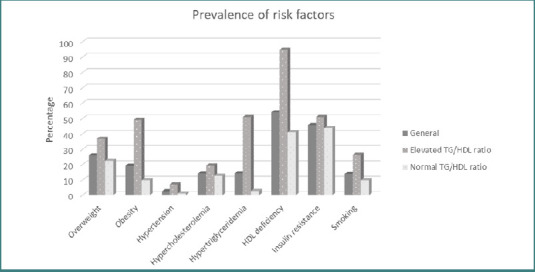
Prevalence of cardiometabolic risk factors. This graphic shows the prevalence of risk factors, divided into the general population and those with elevated TG/HDL ratio and normal TG/HDL ratio.

Figure 1 illustrates the prevalence of various cardiometabolic risk factors. HDL deficiency was the most common risk factor, followed by insulin resistance, overweight, and obesity.

The participants were categorized based on their BMI into normal weight, overweight, and obesity groups, and the analyzed variables were compared among these groups. The results are presented in Table 2. Age, blood pressure, abdominal circumference, triglycerides, TG/HDL ratio, and HOMA index were significantly higher in individuals with obesity compared to those classified as normal weight. HDL levels were significantly lower in the obese group than in the normal weight group. Additionally, abdominal circumference, triglycerides, the TG/HDL ratio, and the HOMA index were significantly higher in the overweight group compared to the normal weight group.

**Table 2:** Comparison of anthropometric and biochemical variables among normal weight, overweight, and obese groups based on BMI

Variable	Normal weight *n* = 131	Overweight *n* = 62	Obesity *n* = 46	*P* value
Age (years)	20.06 ± 1.49	20.41 ± 1.42	21.26 ± 1.90*	0.000
SBP (mmHg)	109.08 ± 9.75	112.22 ± 11.15	121.56 ± 12.07*	0.000
DBP (mmHg)	71.03 ± 8.72	72.50 ± 10.85	79.89 ± 10.70*	0.000
WC (cm)	77.96 ± 7.72	90.79 ± 8.32*	104.35 ± 13.28*	0.000
Glucose (mg/dL)	81.75 ± 7.18	81.91 ± 7.21	82.47 ± 7.30	0.842
Cholesterol (mg/dL)	166.07 ± 27.11	173.24 ± 37.06	166.56 ± 43.98	0.365
Triglycerides (mg/dL)	78.27 ± 31.08	106.30 ± 64.37*	169.39 ± 24.78*	0.000
HDL (mg/dL)	50.32 ± 11.49	44.00 ± 10.70	40.08 ± 14.12*	0.000
TG/HDL	1.66 ± 0.81	2.67 ± 1.76*	4.54 ± 5.74*	0.000
HOMA - IR (mmol/UI)	2.38 ± 1.55	3.36 ± 2.49*	4.38 ± 2.42*	0.000

Mean ± standard deviation. ANOVA, *P* value <0.05 was considered significant. *Difference with respect to the normal weight group. SBP, Systolic Blood Pressure; DBP, Diastolic Blood Pressure; WC, Waist Circumference; HDL, High-Density Lipoprotein; HOMA, Homeostatic Model Assessment.

**Figure 2:**
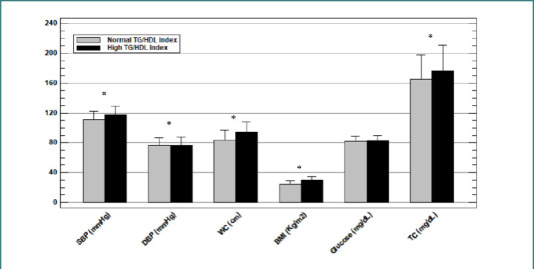
Comparison of anthropometric and biochemical variables according to the TG/HDL ratio. Mean ± SD, Student’s t-test. *P* value <0.05 was considered as significant. SBP, Systolic Blood Pressure; DBP, Diastolic Blood Pressure; WC, Waist Circumference; HDL, High-Density Lipoprotein; TC, Total Cholesterol.

Using a cutoff point of ≥2.5 for women and ≥3.5 for men for the TG/HDL ratio, the population was divided into those with a normal TG/HDL ratio and those with an elevated TG/HDL ratio. Anthropometric and biochemical variables were compared between the two groups. Blood pressure, abdominal circumference, BMI, and total cholesterol were significantly higher in the group with an elevated TG/HDL ratio (Figure 2). The HOMA index was also significantly higher in individuals with an elevated TG/HDL ratio (3.51 ± 2.27) than in those with a normal TG/HDL ratio (2.87 ± 2.09; *P* =0.019).

Table 3 presents the association between TG/HDL ratio and various cardiometabolic risk factors. The analysis shows a significant association with obesity (OR = 27.56; 95% CI, 10.50-75.31; *P* < .001). For abdominal obesity, the odds ratio was 4.41 (95% CI, 2.58-9.30; *P* < .001), and for overweight, it was 9.30 (95% CI, 3.68-23.50; *P* < .001). Smoking had an OR of 3.29 (CI, 1.533 – 7.07; *P* = 0.0022), while a family history of T2DM had an OR of 2.91 (CI, 1.474 – 5.765; *P* = 0.0021).

**Table 3:** Association of TG/HDL ratio with cardiometabolic risk factors

	Odds Ratio	*P* value	Confidence interval (CI)
Insulin resistance (HOMA)	1.38	0.2887	0.758 – 2.25
Arterial hypertension	3.39	0.1197	0.665 – 2.17
Obesity (BMI > 30 Kg/m^2^)	27.56	<0.0001	10.50 – 75.31
Abdominal obesity	4.41	<0.0001	2.587 – 9.30
Overweight (BMI 25.0 - 29.9 Kg/m^2^)	9.30	<0.0001	3.681 – 23.50
Smoking	3.29	0.0022	1.533 – 7.07
Family history of T2DM	2.92	0.0021	1.474 – 5.76

Chi-square test; *P* < 0.05 was considered significant.

A complementary analysis was included to identify whether the pattern of cardiovascular risk factors varied according to age. Due to the narrow age range, we stratified the groups into those aged 18-21 and 22-24, representing the beginning and end of university life, respectively. The comparison can be seen in Table 4, where statistically significant differences were observed in the TG/HDL ratio, BMI, triglycerides, waist circumference, HDL, systolic blood pressure, and diastolic blood pressure. All values were higher in individuals aged 22-24, except for HDL, which was lower in this group. This highlights the importance of early prevention, where risk factors are less pronounced.

**Table 4:** Comparison of anthropometric and biochemical variables according to age

Parameter	18 – 21 years old	22 – 24 years old	*P* value
SBP (mmHg)	111 ± 11.4	116 ± 11.2	< 0.001*
DBP (mmHg)	72.1 ± 9.6	76.7 ± 11.6	0.003*
WC (cm)	85 ± 12	93 ± 16	<0.001*
BMI (Kg/m^2^)	24.8 ± 4.8	28.12 ± 6.24	<0.001*
Glucose (mg/dL)	80.9 ± 6.1	82.1 ± 7.4	0.284
Total cholesterol (mg/dL)	167.9 ± 32	168.39 ± 39	0.937
Triglycerides (mg/dL)	94.1 ± 78	120 ± 68	0.011*
HDL (mg/dL)	48 ± 12	42 ± 11	0.002*
HOMA-IR	2.98 ± 2.14	3.16 ± 2.19	0.601
TG/HDL	2.16 ± 1.79	3.12 ± 2.19	<0.001*

Mean ± standard deviation. Student’s t-test. *P* < 0.05 was considered significant.

## DISCUSSION

The results of this study indicate that the TG/HDL ratio is a significant predictor of cardiometabolic risk in a young population. The observed correlations between higher TG/HDL-C ratios and increased markers of cardiometabolic risk, such as elevated blood pressure, higher waist circumference, and increased HOMA index, underscore the importance of this ratio as an early indicator of potential health issues.

Among the observed parameters, obesity is a known risk factor for morbidity and premature mortality [19]. Chronic conditions such as hypertension, dyslipidemia, and CVD often accompany obesity [20-22]. Our results show a prevalence of 25.9% for overweight and 19.2% for obesity, with rates rising to 36.8% and 49.1%, respectively, in individuals with an elevated TG/HDL ratio. These findings are consistent with the ENSANUT 2022 report, where obesity prevalence among individuals over 19 years old was reported at 41% [23].

Another crucial aspect of this study is insulin resistance, which, along with hyperinsulinemia, triggers early lipid alterations. Insulin resistance promotes hypertriglyceridemia and vice versa, mainly as visceral adipocytes exhibit heightened resistance to insulin [20]. None of the study participants exhibited elevated blood glucose levels, which is understandable given their age and the low frequency of diabetes in this demographic. This is consistent with findings from other studies reporting either no cases or very low frequencies of diabetes and prediabetes in the 15-24 age group [22,24]. However, insulin resistance was prevalent in 45.6% of the study population, as diagnosed by the HOMA index.

Insulin hypersecretion fosters increased fatty acid synthesis, particularly in the liver and adipose tissue [25]. Hyperinsulinemia is also pivotal in metabolic syndrome and may correlate with elevated triglycerides and reduced HDL-cholesterol [26]. Our study suggests that evaluating an elevated TG/HDL ratio enables timely intervention to manage insulin resistance, as well as the prevention and treatment of dyslipidemia in line with therapeutic goals. From a clinical perspective, the TG/HDL ratio could be used as a simple, cost-effective screening tool to identify young individuals at high risk of developing cardiometabolic diseases.

In our study, the TG/HDL ratio was associated with overweight (OR = 9.3), obesity (OR = 27.5), smoking (OR = 3.29), and abdominal obesity (OR = 4.4), and can serve as a secondary marker of insulin resistance (OR = 1.3). A TG/HDL ratio exceeding 2.5 for women and 3.5 for men is proposed to assess the risk of developing insulin resistance in young individuals, correlating with the aforementioned modifiable risk factors.

Published data on the association of this ratio with cardiometabolic risk factors in young people are scarce. Our study demonstrates high sensitivity at the chosen cutoff point, revealing that young individuals with an elevated ratio exhibit a higher risk profile than those with a normal ratio. Thus, alterations in triglycerides and HDL-C, expressed as the TG/HDL ratio, may serve as a predictor of cardiometabolic risk.

Early identification of altered profiles in young individuals should be prioritized to prevent disease progression. Identifying individuals with insulin resistance at risk of developing diabetes can mitigate disease onset. This also allows for timely intervention through lifestyle modifications and, if necessary, pharmacological treatments to mitigate the progression of these conditions.

The complementary analysis performed according to age suggests that the transition from the early to the later years of university life is associated with a deterioration in cardiovascular health markers. The increase in the TG/HDL ratio and other risk factors among the older group indicates an accumulation of lifestyle-related risk factors over a relatively short period.

These age-related differences align with existing literature that underscores the rapid accumulation of cardiometabolic risk during young adulthood. Studies such as Morales *et al*. [27] have shown that lifestyle changes, such as decreased physical activity and poor dietary habits, often occur during university years, contributing to increased cardiometabolic risk. Our findings add to this evidence by providing specific age-related data within a university student population.

From a clinical and public health perspective, these findings underscore the necessity of targeted prevention strategies tailored to young adults. University health programs should prioritize regular screening for cardiometabolic risk factors and promote healthy lifestyle choices through education and support services. Interventions such as promoting physical activity, providing healthy food options on campus, and offering stress management resources could be particularly beneficial. The lifestyle habits acquired during this period will continue into the economically productive life of the individual, making it increasingly difficult to reverse their effects.

As healthcare professionals, it is imperative to promote the prevention of chronic degenerative diseases, especially cardiovascular disease, the leading cause of morbidity and mortality in Mexico. Establishing criteria for early identification of risk factors associated not only with diabetes or cardiovascular disease but also with one of their primary components, insulin resistance, and obesity, is crucial. Early identification will facilitate the prevention of chronic diseases to which they are linked.

Despite the promising results, this study has certain limitations. The cross-sectional nature of the study limits the ability to draw causal inferences. Additionally, the study population may not represent all young individuals, as factors such as ethnicity, socioeconomic status, and genetic predispositions were not fully accounted for. Further longitudinal studies are needed to validate these findings and assess the long-term predictive value of the TG/HDL-C ratio.

Future research should focus on longitudinal studies to establish causality and explore the potential genetic and environmental factors influencing the TG/HDL-C ratio. Moreover, investigating the effectiveness of targeted interventions based on TG/HDL-C ratio screening could provide valuable insights into preventive strategies for cardiometabolic diseases in young populations.

## CONCLUSION

This study reinforces the utility of the TG/HDL-C ratio as a predictive marker of insulin resistance in young individuals and highlights its association with modifiable risk factors for cardiometabolic disease. Adopting this marker in clinical practice could enhance early detection and intervention efforts, ultimately reducing the burden of cardiometabolic diseases.

## Data Availability

Further data is available from the corresponding author upon reasonable request.
